# Editorial: Traditional and innovative approaches for signal detection

**DOI:** 10.3389/fdsfr.2024.1373689

**Published:** 2024-02-06

**Authors:** Marco Tuccori, Andrew Bate, Ugo Moretti, Gianluca Trifirò

**Affiliations:** ^1^ Unit of Adverse Drug Reaction Monitoring, University Hospital of Pisa, Pisa, Italy; ^2^ GlaxoSmithKline (GSK), London, United Kingdom; ^3^ London School of Hygiene and Tropical Medicine, University of London, London, United Kingdom; ^4^ Department of Diagnostics and Public Health, University of Verona, Verona, Italy

**Keywords:** signal detection, signal management, disproportionality analysis, artificial intelligence, natural language processing, real world evidence (RWE), pharmacovigilance

This Research Topic of Frontiers in Drug Safety and Regulation has stimulated animated debate on drug safety signal detection by highlighting two main Research Topic: 1) the need of the community of pharmacovigilance stakeholders for applying a more consistent and robust scientific approach to signal detection; and 2) the implementation of newly developed signal detection methods leveraging the use of the latest generation technologies.

The availability of large scale open-source databases has prompted increased numbers of researchers to carry out studies that often require skills they lack. The principle that applies in general to observational studies also holds true for signal detection in spontaneous reporting adverse drug reaction databases (i.e., having access to data does not translate into knowing how to analyze it). This scenario may lead to spurious results and ultimately incorrect conclusions on benefit-risk profile of drugs which may in turn discourage the use of safe drugs or promote unnecessary additional clinical studies (Suissa, 2008; Bate, 2017).

A strong reminder about the need for adopting high-quality approaches, especially in the context of traditional quantitative analyses for signal detection, comes from the review by Cutroneo et al. This review stems from a discussion among members of the International Society of Pharmacovigilance and in general describes good practices as well as potential criticisms concerning signal detection. This study and similar ones (Khouri et al., 2021; Khouri et al., 2022; Cortes et al., 2023; Khouri et al., 2023) address issues that have been analysed also in the READUS project (Khouri et al., 2023), which brought together a team of international signal detection experts for defining quality standards of scientific publications concerning disproportionality studies in pharmacovigilance. The article by Valdiserra et al. also addresses the importance of and strategy for a robust approach to signal detection, including disproportionality analysis, while considering the wider signal detection continuum of activities. In addition, this study, using the case story of obinutuzumab-associated disseminated intravascular coagulation, highlighted not only the scientific rigor required for the entire signal detection process, but also the importance of collaboration between regulatory agencies and other pharmacovigilance stakeholders. Importantly, clinicians have always played a critical role in the context of signal detection using spontaneous reporting systems (Edwards, 1999). In the era of artificial intelligence (AI) and Big Data-approach It is imperative carefully considering how the constrained time of healthcare professionals involved in the pharmacovigilance routine activities can be best used as a complement to AI, as AI plays an increasing role in operational tasks over time (Trifirò and Crisafulli, 2022). Particularly important is the optimal deployment of PV Physicians, i.e., doctors specialized in the identification, management and minimization of safety problems related to the use of drugs and vaccines, within healthcare systems proposed by Hammad et al. In their article, Hammad et al. also propose an innovative strategy for the assessment of the Bradford Hill criteria that have been historically employed in pharmacovigilance (Meyboom et al., 2002). This system breaks down the complex elements of causality assessment into simple modules (differential diagnosis, identification of evidence of causality, decision making based on an approach specifically tailored based on the type of evidence) and weighs individual and population evidence separately.

Beside the challenges of AI clinical application, as mentioned above, the new frontiers of information technology represented by AI and the tools that it has made available deserve to be commented also in a broader perspective (Wang et al., 2023). We are already beginning to see pharmacovigilance related peer reviewed original research publications on the use of Large Language Models (Dong et al., 2023; Montastruc et al., 2023; Painter et al., 2023). Much research in pharmacovigilance has explored the possible applications of AI and related technologies to make the monitoring of drug safety more efficient. Among the most used tools, natural language processing (NLP) and machine learning play a leading role (Bergman et al., 2023). A key benefit of NLP probably lies in the possibility of mining data from previously unusable sources because they were not codified and supporting the automation of at least some phases of time-consuming analytical processes. Among the benefits of machine learning, there is the possibility of a data driven approach to identifying implicit “rules” within the evaluation process of a signal, which can then be applied by the machine in subsequent evaluations. The study by Lieber et al. offers an interesting example of these methods within the framework of spontaneous reporting. This paper shows how it is possible to optimize the prioritization process by applying NLP to identify useful but uncoded information in the reporting forms and by using algorithms to automate at least part of the process. The results in terms of performance are at this time already promising but it seems inevitable that the technological progress will lead to the achievement of such a high quality of results within a few years as to afford their routine use for this and related applications for pharmacovigilance stakeholders. If we also imagine moving beyond the boundaries of spontaneous reporting, we have known for a few years that real-world drug safety information would be available in not fully coded or even uncoded data sources, ranging from electronic healthcare records (Weiss et al., 2018) to social media (Powell et al., 2022) and beyond (Trifirò et al., 2018). Given the available technology, it will be an ethical imperative to maximize the appropriate use of all available data to generate information useful to protect patient from harm of drugs.

While this Research Topic focusses on two areas of interest, signal detection is a complex and challenging field with many other aspects where active research is being conducted, especially with respect to special population treatments (Sandberg et al., 2020) such as geriatrics/pediatrics, genomics, impact analysis, clinical trial focused signal detection. Use of RWD, i.e., healthcare databases, in signal detection remains a work in progress (Coste et al., 2023) and there is still limited progress on semi-automated multimodal data approaches to signal detection (Coloma et al., 2013; Koutkias and Jaulent, 2015). Practical challenges also remain problems, for examples with ready reuse of benchmarking data. We hope and anticipate continuing to see feverish impactful research activity across all these areas we as a field continue to strive to improve the science of safety signal detection.
